# Factors associated with an unfavorable clinical course in hospitalized patients with pelvic inflammatory disease: a retrospective cohort study of 117 patients from a Japanese academic institution

**DOI:** 10.1186/s12905-022-01925-5

**Published:** 2022-08-17

**Authors:** Naoko Matsuda, Seung Chik Jwa, Saki Tamura, Hiroyuki Suzuki, Masashi Takamura, Akira Namba, Takeshi Kajihara, Ryugo Okagaki, Yoshimasa Kamei, Osamu Ishihara

**Affiliations:** Department of Obstetrics and Gynecology, Saitama Medical University, 38 Morohongo, Moroyama, Iruma-gun, Saitama, 350-0495 Japan

**Keywords:** Body mass index, C-reactive protein, Endometriosis, Tubo-ovarian abscess, Pelvic inflammatory disease, Risk factors

## Abstract

**Background:**

This study aimed to determine the factors associated with an unfavorable clinical course (emergency surgery and/or prolonged hospitalization) in patients requiring hospitalization owing to pelvic inflammatory disease (PID).

**Methods:**

A retrospective study was performed on 117 patients diagnosed with PID who were admitted to our hospital between January 2014 and December 2018. Multivariate regression analysis was conducted to determine the factors associated with emergency surgical intervention, and prolonged hospitalization in a subgroup of successful expectant management (n = 93).

**Results:**

The average age (mean ± standard deviation) of the patients was 41.2 ± 12.5 years; 16 (13.7%) were postmenopausal; 81 patients (69.2%) complicated with a tubo-ovarian abscess (TOA) of which 59 (72.9%) had an ovarian endometrioma; and 19 patients (16.2%) had a history of various intrauterine manipulations. Emergency surgery was performed in 24 patients (20.5%), and patients with TOA underwent emergency surgery more often than did patients without TOA (25.9% vs. 8.3%, *p* = 0.03), and TOA was associated with longer length of hospital stay (17.1 days vs. 8.0 days, *p* = 0.01). Smoking, postmenopausal status, past medical history of PID, and high C-reactive protein (CRP) level at admission were significantly associated with emergency surgery. In patients with successful expectant management, obesity (body mass index ≥ 30) and high WBC and CRP level at admission were significantly associated with prolonged hospitalization.

**Conclusions:**

Of the patients requiring hospitalization owing to PID, TOA was associated with both emergency surgery and prolonged hospital stay. Patients with increased inflammatory markers and obesity should be considered to be at a high risk for unfavorable clinical course in the management of PID.

## Background

Pelvic inflammatory disease (PID) is an acute infection of the upper genital tract involving the uterus, oviduct, and ovaries, and is usually initiated by a sexually transmitted agent that ascends from the cervix to the pelvis, such as *Chlamydia trachomatis* and *Neisseria gonorrhoeae* [1–3]. Transcervical intrauterine manipulations, such as removal of an intrauterine device (IUD), endometrial sampling, infertility assessment with hysterosalpingography, and embryo transfer, are also known risk factors for PID [2, 4–6]. Most patients diagnosed with PID can be treated with oral antibiotic agents without hospitalization [7]. However, in those with severe PID, hospitalization and antibiotic therapies are required, and sometimes, surgical intervention is necessary to drain the pelvic abscess and/or remove the sources of infection, such as the uterus and adnexa.

To date, several factors, such as intrauterine manipulation, serum inflammatory markers, and size of the ovarian abscess, have been associated with poor clinical course in patients with PID [8–11]. However, most studies are based on a limited sample size of < 100, and available evidence is very limited. Furthermore, even in patients with successful expectant management, factors associated with prolonged hospitalization have not been well elucidated.

Thus, the current study aimed to determine the factors associated with poor clinical course (emergency surgery and prolonged hospitalization) in a large sample (i.e. more than 100 patients) who required hospitalization owing to PID.

## Methods

This is a retrospective study based on a medical chart review at Saitama Medical University. Patients diagnosed with PID who required hospitalization in our institution between January 2014 and December 2018 were considered eligible for the study. Diagnosis of PID in this study was based on the U.S. Centers for Disease Control and Prevention (CDC) sexually transmitted diseases treatment guidelines [3]. There were 155 patients who were initially diagnosed with PID and required hospitalization during the study period. Among these, the following patients were excluded from this study: 12 patients who were later diagnosed with torsion of ovarian cyst; 7 patients with other inflammatory diseases, such as appendicitis; 2 patients did not meet the criteria for PID by the CDC guidelines; 5 patients with postoperative infection within 1 month; 5 patients owing to cancer; and 5 patients who were pregnant or in puerperium. In addition, 1 patient who had complications with multiple organ failure at admission and died within 24 h and 1 patient who declined hospitalization were excluded. Finally, the remaining 117 patients were included in the analysis. This study was approved by the Institutional Review Board of Saitama Medical University Hospital (approval no. 19054.01).

We investigated patients’ background information, including age; parity; body mass index (BMI); smoking status; menopausal status; sexual intercourse before the onset of PID; medical complications, such as diabetes mellitus; and gynecologic conditions, such as uterine fibroma, endometriosis, and adenomyosis. Gynecologic conditions were diagnosed by either ultrasound or magnetic resonance imaging. We also reviewed the history of intrauterine manipulations, such as endometrial sampling, removal of IUD, infertility assessment with hysterosalpingography, and embryo transfer. Moreover, Fever at admission (≧38.0 °C), tubo-ovarian abscess (TOA) and laboratory tests for total white blood cell count (WBC) and C-reactive protein (CRP) level at admission were included in the analysis.

The main outcome of this study was the requirement of surgical intervention, including abdominal drainage, adnexectomy, and hysterectomy, by either laparotomy or laparoscopy. Additionally, in patients with successful expectant management using antibiotics, duration of hospitalization were analyzed as secondary outcome. For expectant management, a majority of patients received empirical administration of cefmetazole sodium and of either metronidazole or minocycline hydrochloride.

For the 117 patients, we calculated crude odds ratio (OR) and 95% confidence interval (95% confidence interval, CI) of each factor for the outcome of emergency surgery using bivariate logistic regression. Next, adjusted ORs (aORs) and 95% CIs were calculated using forward stepwise variable selection methods considering the effect of confounding factors. In patients with successful expectant management (n = 93), multivariate linear regression analysis was performed using the same strategy for variable selection to investigate the association between risk factors and duration (i.e. days) of hospitalization. A two-tailed *p* value of < 0.05 was considered statistically significant. All analyses were performed using the STATA MP statistical package version 16.1 (Stata, College Station, TX, USA).

## Results

Baseline characteristics of the sample population are summarized in Table 1. Among the 117 analyzed samples, 16 (13.7%) were from postmenopausal women. There were 22 patients (18.8%) who were overweight (BMI 25–30), and 15 (12.8%) were obese (BMI ≥ 30). A majority of patients (n = 81, 69.2%) had a tubo-ovarian abscess (TOA), of whom 59 (72.9%) had an ovarian endometrioma. Nineteen patients (16.2%) had a history of intrauterine manipulation.

**Table 1:** Baseline characteristics of sample population (n = 117)

	Mean ± SD or n (%)
Age (years)	41.2 ± 12.5
Parity	
0	58 (49.6)
≥ 1	59 (50.4)
BMI (Kg/m^2^)	23.7 ± 5.0
< 18.5	11 (9.4)
18.5–25	69 (59.0)
25–30	22 (18.8)
≥ 30	15 (12.8)
Smoking	15 (12.8)
Post-menopause	16 (13.7)
Past medical history of PID	21 (18.0)
DM	3 (2.6)
Gynecological conditions	
Uterine fibroma	37 (31.6)
Endometriosis	63 (53.9)
Adenomyosis	19 (16.2)
Intrauterine manipulation	19 (16.2)
Endometrial biopsy	9 (7.7)
Removal of IUD	2 (1.7)
Embryo transfer	6 (5.1)
Hysterosalpingography	2 (1.7)
Positive for PCR testing for Chlamydia trachomatis^a^	4 (5.1)
Sexual intercourse before the onset of PID	9 (7.7)
Fever (≧38.0 °C) at admission	43 (36.8)
Tubo-ovarian abscess	81 (69.2)
Bilateral	20 (24.7)
Size of abscess, (cm)	5.8 (1.5)
WBC at admission, (× 10^3^/μL)	14.6 ± 5.2
CRP at admission, (mg/dL)	12.0 ± 9.6

^a^78 patients were tested for DNA-PCR of Chlamydia trachomatis

*BMI* body mass index, *CRP* C-reactive protein, *DM* diabetes mellitus, *IUD* intrauterine device, *PID* pelvic inflammatory disease, *SD* standard deviation, *WBC* white blood cell count

The proportion of patients who underwent emergency surgery and duration of hospitalization with or without TOA are presented in Table 2. Emergency surgery was performed in 24 patients (20.5%), of which 21 (87.5%) were in patients with TOA. The proportion of emergency surgery in patients with TOA was more than three-fold (25.9%) in those without TOA (8.3%) with statistical significance (*p* = 0.03). Of the 93 patients with successful expectant management, the mean duration of hospitalization was 8.3 days (SD = 5.3) in patients without TOA and 9.4 days (SD = 4.6) in patients with TOA with no statistical difference (*p* = 0.31).

**Table 2:** Surgical intervention and duration of hospitalization stratified by tubo-ovarian abscess

	Without TOA (n = 36)	With TOA (n = 81)	*p* value
Emergency surgery	3 (8.3)	21 (25.9)	0.03
Hospital length of stay, (days)			
Emergency surgery (n = 24)	8.0 ± 2.0	17.1 ± 5.6	0.01
Successful expectant management (n = 93)	8.3 ± 5.3	9.4 ± 4.6	0.31

Data are shown as mean ± SD or n (%)

TOA, tubo-ovarian abscess

Crude and aORs of risk factors for emergency surgery are shown in Table 3. In crude analysis, TOA and CRP level at admission were significantly associated with emergency surgery. In the adjusted model, CRP level at admission, smoking status, postmenopausal status, and past medical history of PID were significantly associated with emergency surgery. + 1 mg/dL increase in CRP at admission was associated with significantly increased adjusted odds for emergency surgery (aOR = 1.07, 1.02 to 1.13, *p* = 0.01).

**Table 3:** Crude and adjusted odd ratios of risk factors for emergency surgery (n = 117)

	Crude OR (95% CI)	*p* value	Adjusted OR	*p* value
Age (years)	1.03 (0.998 to 1.07)	0.07	–	–
Parity				
0	Reference		–	–
≥ 1	2.33 (0.91 to 5.96)	0.08	–	–
BMI				
< 18.5	0.96 (0.18 to 4.97)	0.96	–	–
18.5–25	Reference		–	–
25–30	2.01 (0.68 to 5.93)	0.21	–	–
> 30	0.66 (0.13 to 3.30)	0.62	–	–
Smoking	2.18 (0.67 to 7.13)	0.20	**4.98 (1.17 to 21.2)**	**0.03**
Post-menopause	2.77 (0.89 to 8.59)	0.08	**5.66 (1.41 to 22.8)**	**0.02**
Past medical history of PID	2.32 (0.81 to 6.62)	0.12	**3.99 (1.10 to 14.5)**	**0.04**
DM	1.98 (0.17 to 22.8)	0.58	–	–
Uterine fibroma	0.86 (0.32 to 2.31)	0.77	–	–
Endometrioma	0.82(0.34 to 2.02)	0.67	–	–
Adenomyosis	1.04 (0.31 to 3.48)	0.95	–	–
Intrauterine manipulation	2.05 (0.69 to 6.13)	0.20	3.27 (0.90 to 11.8)	0.07
Sexual intercourse before the onset of PID	0.46 (0.05 to 3.88)	0.48	–	–
Fever (≧38.0 °C)	0.65 (0.25 to 1.73)	0.39	–	–
Tubo-ovarian abscess	**3.85 (1.07 to 13.9)**	**0.04**	3.58 (0.90 to 14.2)	0.07
WBC at admission	0.994 (0.91 to 1.09)	0.90	–	–
CRP at admission	**1.06 (1.01 to 1.11)**	**0.02**	**1.07 (1.02 to 1.13)**	**0.01**

*BMI* body mass index, *CI* confidence interval, *CRP* C-reactive protein, *DM* diabetes mellitus, *OR* odds ratio, *PID* pelvic inflammatory disease, *WBC* white blood cell count

Significantly increased or reduced odds are indicated by boldface

In patients with successful expectant management (n = 93), coefficient of risk factors for duration of hospitalization is presented in Table 4. In crude analysis, age, BMI ≥ 30, adenomyosis, WBC count, and CRP level at admission were significantly associated with prolonged hospitalization. In the multivariate model, BMI ≥ 30, high WBC and CRP level at admission were significantly associated with prolonged hospitalization, whereas postmenopausal status was significantly associated with shorter duration of hospitalization. Compared with patients with normal BMI (18.5–25), patients with obesity (BMI ≥ 30) demonstrated significantly longer hospitalization (+ 2.68 days, 95% CI, 0.09–5.26, *p* = 0.04) among patients with successful expectant management. Similarly, + 1 mg/dL increase in CRP at admission was associated with significantly longer hospitalization (+ 0.19 days, 95% CI, 0.092–0.29, *p* < 0.001).

**Table 4:** Coefficient of risk factors for duration of hospitalization among patients with successful expectant management (n = 93)

	Coefficient (95% CI)	*p* value	Adjusted coefficient	*p* value
Age (years)	**0.11 (0.03 to 0.19)**	**0.008**	**0.14 (0.05 to 0.24)**	**0.004**
Parity				
0	Reference		–	–
≥ 1	0.10 (− 1.90 to 2.11)	0.92	–	–
BMI				
< 18.5	− 0.93 (− 4.28 to 2.41)	0.58	0.40 (− 2.55 to 3.35)	0.79
18.5–25	Reference		Reference	
25–30	1.73 (− 0.98 to 4.44)	0.21	0.88 (− 1.58 to 3.35)	0.48
> 30	**3.66 (0.78 to 6.53)**	**0.01**	**2.68 (0.09 to 5.26)**	**0.04**
Smoking	− 1.31 (− 4.52 to 1.91)	0.42	–	–
Post-menopause	0.71 (− 2.51 to 3.93)	0.66	**− 3.76 (− 7.51 to − 0.009)**	**0.049**
Past medical history of PID	0.54 (− 2.25 to 3.33)	0.70		–
DM	3.61 (− 3.24 to 10.5)	0.31	–	–
Uterine fibroma	1.97 (− 0.13 to 4.06)	0.07	–	–
Endometrioma	1.07 (− 0.93 to 3.07)	0.29	–	–
Adenomyosis	**2.82 (0.17 to 5.47)**	**0.04**	–	–
Intrauterine manipulation	0.48 (− 2.39 to 3.36)	0.74	–	–
Sexual intercourse before the onset of PID	− 2.56 (− 6.09 to 0.96)	0.15	–	–
Fever (≧38.0 °C)	1.64 (− 0.38 to 3.66)	0.11	–	–
Tubo-ovarian abscess	1.08 (− 1.0 to 3.15)	0.31	–	–
WBC at admission	**0.34 (0.16 to 0.51)**	** < 0.001**	**0.18 (0.009 to 0.36)**	**0.04**
CRP at admission	**0.21 (0.10 to 0.31)**	** < 0.001**	**0.19 (0.092 to 0.29)**	** < 0.001**

*BMI* body mass index, *CI* confidence interval, *CRP* C-reactive protein, *DM* diabetes mellitus, *OR* odds ratio, *PID* pelvic inflammatory disease, *WBC* white blood cell count

Significantly increased or reduced coefficients are indicated by boldface

## Discussion

In this study investigating the factors associated with an unfavorable clinical course among 117 patients requiring hospitalization owing to PID, complications of TOA, smoking, postmenopausal status, past medical history of PID, and high CRP level at admission were significantly associated with increased risk of emergency surgery; among patients with successful expectant management, high WBC and CRP levels at admission were significantly associated with prolonged hospitalization. Notably, we found that obesity (BMI ≥ 30) was significantly associated with increased risk of prolonged hospitalization in successful expectant management.

Our study demonstrated that relatively large proportion of patients with PID requiring hospitalization were postmenopausal (13.7%). Generally, PID is dominant in younger, sexually active population, and it is often caused by sexually transmitted infectious agents, such as *C. trachomatis* and *N. gonorrhoeae* [2]. In our study, however, only 5.1% of patients had sexually transmitted infectious disease. Thus, we believe PID is now critical in postmenopausal women. A recent systematic review investigating the risk factors for TOA in postmenopausal women reported that the prevalence of postmenopausal status in the total TOA cases was 6%–18%. Endometrial sampling (6–45%) and removal of a longstanding IUD (31%–50%) were significant risk factors for postmenopausal women [5]. Postmenopausal women tend to receive less conservative, more interventional treatment [5, 10]. Our study also demonstrated that 37.5% of the postmenopausal patients underwent emergency surgery, accounting for almost double of the premenopausal patients (17.8%). This might be attributed to delayed diagnosis owing to their atypical presentation and symptoms and the physician’s decision of not preserving the reproductive function. In fact, postmenopausal patients with successful expectant management had significantly shorter duration of hospitalization in the adjusted analysis, which reflects that less severe cases remained for expectant management rather than premenopausal patients (Table 4).

In our study, more than half (53.9%) of the patients had endometriosis as a gynecologic condition and of the 81 patients with TOA, 72.9% were derived from ovarian endometrioma, suggesting that endometriosis is a highly important background factor in the development and pathogenesis of PID. The reason why endometriosis is more common in patients with PID is because many patients with complicated endometriosis present with infertility, and intrauterine manipulations, such as hysterosalpingography and embryo transfer, may contribute to the development of PID. A recent study investigating predictive factors for emergency surgery for PID in 22 patients with ovarian endometrioma reported that intrauterine or intrapelvic procedure before the onset of PID was significantly associated with emergency surgery (crude OR = 8.33; 95% CI, 1.02–181.3; *p* = 0.048) [8]. Especially, intrauterine manipulation as infertility treatment among patients with endometriosis should be considered; one study from Israel demonstrated that among 148 hospitalized patients with PID, patients with endometriosis were more likely to have undergone infertility treatment than those without endometriosis [12]. Furthermore, their study reported that patients with endometriosis had more severe clinical courses than those without endometriosis. Our study also demonstrated that 50.0% (2/4) of patients underwent embryo transfer, and 100% (2/2) of these underwent hysterosalpingography complicated with endometriosis and resultant TOA. Thus, we need to be careful regarding PID when patients with infertility having endometriosis undergo intrauterine manipulation, and prophylactic antibiotics may be considered at the time of such procedures.

Similarly, our study found that a large proportion of patients with PID (81 patients, 69.2%) also had TOA. The high rate of TOA may reflect the severity of PID; our study only included patients with PID who required hospitalization, and patients who were cured with oral antibacterial treatment as outpatients were not included. A similarly high rate of TOA in patients with PID (54%) who required hospitalization had been previously reported [13]. TOA is one of the most serious complications of PID, which may cause infertility, ectopic pregnancy, and pelvic thrombophlebitis, and other various complications [15]. In fact, our study demonstrated a high rate of emergency surgery in patients with TOA (25.9%). Thus, caution should be exercised when managing patients with TOA.

High CRP level at admission was associated with an increased risk of both emergency surgery and prolonged hospitalization among patients with successful expectant management, which was consistent with the findings of previous studies. Terao et al. investigated predictive factors for poor clinical course (> 7 days of hospitalization and/or surgery) in 93 patients with PID and reported that high CRP level at admission was independently associated with poor clinical course [9]. They revealed that the area under the curve of CRP level was 0.67, and the cutoff value of 4.4 mg/dL demonstrated a sensitivity of 76.2% and specificity of 58.4% for poor clinical course. Considering our results, high CRP level at admission would be an important predictor for emergency surgery and prolonged hospitalization even in successful expectant management.

Notably, our study demonstrated that obesity (BMI ≥ 30) was significantly associated with increased risk of prolonged hospitalization in successful expectant management. To the best of our knowledge, there have been no studies that demonstrated the association between obesity and prolonged hospitalization in successful expectant management of PID. One study from Singapore investigating risk factors for failed conservative management in 136 patients with TOA reported that BMI was significantly associated with failed conservative management (aOR for every kg/m^2^ increase in BMI, 1.10; 95% CI, 1.00–1.20; *p* = 0.04) [11]. Several pathophysiological reasons in obese patients would be suggested; obesity has been reported to reduce immunological response by producing proinflammatory factors by adipose tissue and altered T-cell function [13, 14]. Furthermore, obesity can cause various physiological changes that lead to altered pharmacokinetic and pharmacodynamic conditions for antibiotic therapy [15]. Thus, the clinician should consider obese patients to be at a high risk for unfavorable clinical course, and careful strategy for dose selection, such as selecting higher end of dose range using therapeutic drug monitoring, might be effective in the treatment of patients with obesity.

Although this is the first study to elucidate the association between obesity and prolonged hospitalization in patients with PID, it has several limitations. First, this is a retrospective observational study, which has a high risk of introducing the possibility of confounding and biases in the study results. The indication for emergency surgery may vary across patients; in fact, postmenopausal patients tend to undergo emergency surgery rather than expectant management. In many cases, the time of onset could not be elicited; therefore, the relationship between the time of onset and start of antibiotic use could not be examined. Further, because of the imprecise nature of patient-recalled information, for long-term users of IUDs, no valid data regarding duration of use were available. In addition, because many of the cases were emergencies, detailed imaging evaluation was not possible, making it impossible to obtain accurate information on clinical events such as tumor rupture. Similarly, vaginal cultures and abscess or blood cultures could not be analyzed because of the high proportion of cases with missing data, although they were performed in some cases. Unmeasured confounders such as patients’ socioeconomic status may also affect the study results. Thus, to confirm our findings, further study, particularly a prospective study, to investigate risk factors for poor prognosis among hospitalized patients with PID is essential.

## Conclusions

In conclusion, our study demonstrates that 14% of patients requiring hospitalization owing to PID were postmenopausal and almost 70% of patients complicated with TOA mostly derived from ovarian endometrioma. Furthermore, smoking, postmenopausal status, past medical history of PID, and high CRP level at admission were significantly associated with emergency surgery, and high WBC and CRP level at admission and obesity (BMI ≥ 30) were significantly associated with prolonged hospitalization even with successful expectant management. Based on our findings, patients with obesity and elevated WBC and CRP level should be considered to be at a high risk for poor clinical course in the treatment of PID.

## Data Availability

The datasets generated and/or analysed during the current study are not publicly available because the data used for this study includes personal information but are available from the corresponding author on reasonable request.

## Abbreviations

BMI: Body mass index
CDC: Centers for disease control and prevention
CI: Confidence interval
CRP: C-reactive protein
IUD: Intrauterine device
OR: Odds ratio
PID: Pelvic inflammatory disease
SD: Standard deviation
TOA: Tubo-ovarian abscess
WBC: White blood cells
